# The antidepressant fluoxetine acts on energy balance and leptin sensitivity via BDNF

**DOI:** 10.1038/s41598-018-19886-x

**Published:** 2018-01-29

**Authors:** Gaia Scabia, Ilaria Barone, Marco Mainardi, Giovanni Ceccarini, Manuela Scali, Emma Buzzigoli, Alessia Dattilo, Paolo Vitti, Amalia Gastaldelli, Ferruccio Santini, Tommaso Pizzorusso, Lamberto Maffei, Margherita Maffei

**Affiliations:** 1 Obesity Center at the Endocrinology Unit, Department of Clinical and Experimental Medicine, Via Paradisa 2, 56126 Pisa, Italy; 2 Dulbecco Telethon Institute, Via Varese, 16B, Rome, 00185 Italy; 3 Laboratory of Neurobiology, Scuola Normale Superiore, Piazza dei Cavalieri 7, 56100 Pisa, Italy; 4 Institute of Clinical Physiology, Italian National Research Council, Via Moruzzi 1, 56124 Pisa, Italy; 5 Institute of Neuroscience, Italian National Research Council, Via Moruzzi, 56124 Pisa, Italy; 6 Dipartimento NEUROFARBA, Università di Firenze, via di San Savi, 26, 50235 Firenze, Italy; 7 Present Address: Laboratory of Neurobiology, Scuola Normale Superiore, Piazza dei Cavalieri 7, 56100 Pisa, Italy

## Abstract

Leptin and Brain Derived Neurotrophic Factor (BDNF) pathways are critical players in body weight homeostasis. Noninvasive treatments like environmental stimulation are able to increase response to leptin and induce BDNF expression in the brain. Emerging evidences point to the antidepressant selective serotonin reuptake inhibitor Fluoxetine (FLX) as a drug with effects similar to environmental stimulation. FLX is known to impact on body weight, with mechanisms yet to be elucidated. We herein asked whether FLX affects energy balance, the leptin system and BDNF function. Adult lean male mice chronically treated with FLX showed reduced weight gain, higher energy expenditure, increased sensitivity to acute leptin, increased hypothalamic BDNF expression, associated to changes in white adipose tissue expression typical of “brownization”. In the Ntrk2^tm1Ddg^/J model, carrying a mutation in the BDNF receptor Tyrosine kinase B (TrkB), these effects are partially or totally reversed. Wild type obese mice treated with FLX showed reduced weight gain, increased energy output, and differently from untreated obese mice, a preserved acute response to leptin in terms of activation of the intracellular leptin transducer STAT3. In conclusion, FLX impacts on energy balance and induces leptin sensitivity and an intact TrkB function is required for these effects to take place.

## Introduction

The hormone leptin, secreted by the white adipose tissue (WAT), plays a key role in the complex interplay between peripheral organs and the brain to regulate body weight^1^, by informing the brain about the status of long-term energy stores, in order to repress food intake and promote energy expenditure^2^. The primary sensor of leptin is the arcuate nucleus (ARC) of the hypothalamus^3^, which expresses the active form of the leptin receptor, OBRb^4^ that mainly signals through the Janus Kinase 2/Signal Transducer and Activator of Transcription 3 (JAK2/STAT3) pathway. OBRb is expressed by two neuronal sensors of leptin, identified according to the expression of ProOpioMelanoCortin (POMC) and Neuropeptide Y (NPY) respectively^5^. The former is activated by leptin and induces an anorectic response^6^, whereas the latter, inhibited by leptin, stimulates feeding^7^. The above depicted scenario is how the system should work to regulate energy balance and prevent excessive weight accumulation. However, obese people display high levels of leptin and therapy with the recombinant hormone has proved largely ineffective to treat obesity^8^, leading to hypothesize the existence of a set-point for leptin responsiveness, which is programmed via an interaction between genetic background and environment^9^. Obesity can be interpreted as an upwards shift of the set point, resulting in a positive energy balance that ultimately leads to fat stores increased deposition. Modulating leptin sensitivity, rather than leptin levels, is then an emerging frontier for obesity therapy and prevention. This aim has been successfully pursued using various experimental strategies including genetic deletion of negative regulators of OBRb function like suppressor of cytokine signaling 3 (SOCS3) and Protein Tyrosine Phosphatase 1B (PTP1B)^10,11^, selective stimulation of neuronal populations^12^ and local hypothalamic infusion of the anorectic peptide amylin^13^. We and others previously demonstrated that in alternative to these approaches, the set-point for leptin sensitivity can be reprogrammed in rodents by changing their “life style”, achieved by means of physical exercise^14^ or environmental enrichment (EE), a model in which physical as well as cognitive and social activities are promoted. EE results in lower plasmatic leptin and higher phosphorylation of STAT3 in the ARC, which correlate with increased expression of Brain Derived Neurotrophic Factor (BDNF)^15^. BDNF, a master regulator of neural plasticity^16–18^, plays a critical role in energy homeostasis: classical studies showed that BDNF infusion results in diminished appetite and body weight^19^, whereas mice haploinsufficient for BDNF become hyperphagic and obese^20,21^. Consistently, mice with diminished expression of the gene coding for the BDNF receptor Tyrosine kinase B (TrkB) show increased food intake and body weight^22^. In humans, similar alterations were linked to BDNF haploinsufficiency^23^ and to a *de novo* missense mutation in the TrkB gene^24^.

BDNF expression can be induced in the brain by Fluoxetine (FLX), a Selective Serotonin Reuptake Inhibitor (SSRI) currently in use for treating major depression^25,26^, and emerging as an interesting tool to manipulate neuronal plasticity in various brain areas, with outcomes similar to EE^27,28^; indeed, FLX is able to enhance neurogenesis and neuronal turnover in the hippocampus^29^, to reinstate juvenile-like plasticity in the adult rat visual cortex^30^, and to improve recovery of spinal cord injuries^31^.

FLX has been employed in the obesity therapy in the past with controversial results. Typically, the therapy was effective in the first weeks of treatment but weight was regained afterwards^32^ and no comprehensive understanding of the underlying molecular mechanisms of its action on energy balance has been achieved.

In the present study we asked whether the leptin system and the BDNF axis were modified by FLX and whether this could explain its effect on energy homeostasis.

We found that FLX treatment of adult mice reduces weight gain both in normal conditions and upon obesity which correlate with higher energy expenditure, increased response to acute leptin and higher hypothalamic BDNF. In the absence of an intact BDNF pathway most of these effects are attenuated or lost.

## Materials and Methods

### Animal care

All animal care protocols and procedures were approved by the Italian Ministry of Health (protocol 108/2015-PR) and all experiments were performed in accordance with relevant guidelines and regulations. Mice were maintained on a 12 hours light/dark cycle in a temperature controlled animal facility, with *ad libitum* access to water and standard chow food diet (CFD, Lab Diet 5010, calories provided by protein = 28.7%, by fat = 12.7%, and by carbohydrate = 58.5%) or high-fat diet (HFD, Bioserve, F3282, calories provided by protein = 19%, by fat = 36%, and by carbohydrate = 35%). Body weight was monitored once a week and food intake was measured every other day.

### Mouse strains

We employed a total of 80 C57BL6/J and 25 transgenic Ntrk2^tm1Ddg^/J male mice carrying a floxed F616A mutation at the locus coding for the BDNF receptor Ntrk2 (neurotrophic tyrosine kinase receptor, type 2; also called TrkB). In this mouse BDNF signaling is effectively blunted by administration of the commercially available compound 1NaPP1^33^, a drug able to cross the blood brain barrier^34^.

We employed male mice since they are more susceptible to become obese upon high fat feeding^35^ and to develop leptin resistance^36^. Further, metabolic parameters of male are more stable with respect to female mice, not being affected by the oestrus cycle.

### Treatments

Fluoxetine (FLX, Galeno s.r.l.) was dissolved in drinking water (0.166 g/L, corresponding to a mean value of 14.2 ± 1.6 and 10.8 ± 1.7 mg/Kg/day per animal for CFD and HFD respectively; the difference in FLX dosage between the two diet regimens is not statistically significant. FLX was prepared fresh every second day. This regimen of treatment follows drug concentrations previously used^30^ and is sufficient to minimize stress^37^. FLX treatment was conducted in 3 different experimental settings: 1. in adult lean mice: 2-months old mice were treated with FLX for 3 weeks, from postnatal day (p) 60 to p81 (FLX-CFD group). The control group (H_2_O-CFD) drunk water during all treatment period. 2. In adult obese mice: starting from weaning (3 weeks of age), mice were fed with HFD for 8 weeks. During the last two weeks of HFD (from 6th to 8th) FLX treatment overlapped with HFD feeding (FLX-HFD group). Before the start of FLX treatment, mice that did not reach 40 grams of body weight were excluded from the analysis. Control group (H_2_O-HFD) received water during all treatment period. 3. In adult mice genetically modified to induce blunted TrkB function: osmotic minipumps (Alzet, model 1004) were filled with 1NaPP1 (Cayman Chemicals) at a concentration of 12 μg/μl (1NaPP1 group), to obtain the delivery of about 100 nmoles/day per mouse of 1NaPP1, or with vehicle (20% DMSO, 2% Tween 20 (Sigma Aldrich)) (VEH group). Ntrk2^tm1Ddg^/J mice were then implanted in the scapular region with one or the other type of osmotic minipumps. After recovery (2 days) 4 groups of mice were generated: treated with FLX for three weeks and receiving or not 1NaPP1 (1NaPP1-FLX and VEH-FLX groups); untreated and receiving or not 1NaPP1 (1NaPP1-H_2_O and VEH-H_2_O groups).

### Plasma leptin

Blood samples were collected at sacrifice by cardiac puncture after an overnight fast. Blood was collected in EDTA tubes and centrifuged in a refrigerated microfuge. Plasma was immediately stored at −20 °C for subsequent assays. Plasma leptin was measured using Mouse/Rat Leptin Quantikine ELISA Kit (R&D Systems).

### Quantitative Real Time PCR

Total RNA was extracted from frozen tissues using TRIzol (Ambion). cDNA was synthetized from 1 µg of total RNA using iScript™ Reverse Transcription Supermix for RT-qPCR (BioRad). Quantitative real-time PCR (RT-PCR) was carried out using iTaq™ Universal Probes Supermix (BioRad) with CFX96TM Real-Time System (BioRad) instrument, and the relative amount of mRNA for *OBRb*, *BDNF*, *CIDEA*, *Prdm16*, *UCP1*, *LPL* and *FAS* genes (Applied Biosystem) was calculated as fold increase on TATA Binding Protein gene (*TBP*) expression (internal control). Data were obtained using ΔΔ−Ct method.

### Leptin sensitivity experiments

At the end of treatment leptin sensitivity of lean mice, obese mice and Ntrk2^tm1Ddg^/J mice was assessed. One hour before dark onset food was removed. At dark onset (6 PM) mice were individually caged and given weighed chow food. Food intake was measured 14 and 24 hours later in basal conditions, when mice were i.p. injected with recombinant mouse leptin (3 mg/Kg body weight, AstraZeneca). 14 hours after leptin injection, food intake was assessed to evaluate the response to exogenous leptin administration. Food intake measurements in the basal conditions were used to calculate percent change from the mean of the baseline.

### Measurements of spontaneous locomotor activity

Opto M3 multi-channel activity monitors (Columbus Instruments, OH, USA) were used to quantify spontaneous horizontal activity of animals. The assessment was conducted in the same conditions of animal facility housing; all measurements were performed from 6:00 PM to 6:00 AM (dark phase) and to 6:00 AM to 6:00 PM (light phase), using animals maintained on a 12 hours light/dark cycle. During the last week of FLX treatment mice were individually caged in cages flanked by Opto M3 monitors. Spontaneous locomotor activity was calculated from infrared beam breaks by determining activity at 1 min intervals. The mean of hourly activity was calculated based on the sum of beam brakes per minute.

### Total energy expenditure (TEE) assessment by Doubly Labeled Water (DLW) method

DLW involves the administration of water labelled with stable isotopes of oxygen (^18^O) and hydrogen (^2^H), i.e., doubly labelled water^38^. After administration, the DLW rapidly equilibrates with the body water (in about 3 hours) and then slowly is cleared. The elimination of H_2_^18^O and ^2^H_2_O from the body water pool occurs at different rates: the hydrogen label is eliminated primarily via outflow of water from the body water pool, while the oxygen label is eliminated both by water loss and from expiration of respiratory CO_2_. The difference between the elimination rates of these two isotopes therefore provides an estimation of the rate of CO_2_ production (rCO_2_), and hence respiration, in an individual over a known time period, that in our case was 72 hours.

Animals were i.p. injected with 270 µl 10%^18^O (Isotec) and 27 µl di 99 %^2^H_2_O and drinking water was removed from the cage for the first 3 hours after injection to avoid dilution of isotopes. Blood samples were collected from the tail vein at 3 (baseline), 48 and 72 hours from injection. 10 µl of plasma were diluted with 190 µl of H_2_O and samples were then analyzed with an Off-Axis Integrated Cavity Output Spectroscopy (OA-ICOS) laser absorption spectrometer (Los Gatos Research (LGR) Liquid Water Isotope Analyzer (LWIA-24d)) that was previously validated *versus* the conventional isotope ratio mass spectrometer^39^. The instrument allows the simultaneous analysis of the 2 H/1 H and 18 O/16 O enrichment in liquid samples (i.e. diluted plasma), with the advantage of being simple and rapid (approximately 25 samples per hour), requiring no decolorizing or distillation steps. Each sample was injected 10 times and the enrichment was calculated as the mean of the last 5 injections to exclude any memory confounding effect deriving from previous injections. A standard curve with increasing known enrichment of both ^18^O and ^2^H was prepared fresh and injected with each batch. Tracer enrichment was calculated as tracer-to-tracee ratio as previously reported^38^.

Total energy expenditure observed during the 72 h of observation was calculated using the following formula:

$$\begin{array}{c}{{\rm{rCO}}}_{{\rm{2}}}=(V/2)\,({\rm{k18}}-{\rm{k2}})\\ {\rm{TEE}}({\rm{kcal}}/{\rm{day}})=(3.815\,({{\rm{rCO}}}_{2}/{\rm{RQ}})+1.232{{\rm{rCO}}}_{2})\ast 1000\end{array}$$

where k18 and k2 are the slopes of the log transformed curve, V is the water volume calculated as Dose of DLW in mmol/intercept of the slope and RQ is the respiratory quotient.

### Histological analysis

STAT3 activation in response to acute leptin injection was performed as previously described^15,40,41^. Animals were fasted for 2 hours before receiving i.p. leptin at a dose of 3 mg/kg; 45 min later, mice were anesthetized with an overdose of chloral hydrate. They were then transcardially perfused with PBS, followed by 4% (vol/vol) paraformaldehyde in 0.1 M phosphate buffer (PB). Brains were postfixed in the same fixative at 4 °C for 4 hours and then sunk in 30% (weight/vol) sucrose in 0.1 M PB at 4 °C. Brains were then frozen in isopentane and stored at −80 °C. Fifty-micrometer-thick coronal sections comprising the ARC were cut on a microtome (Leica) and processed for immunofluorescence. For STAT3 and pSTAT3 immunofluorescence (IF), sections were permeabilized in 100% (vol/vol) methanol at 4 °C for 5 min before blocking and were incubated overnight at 4 °C with STAT3 primary antibodies (1:250 rabbit anti-mouse, Cell Signaling Technologies; #12640) or pSTAT3 primary antibodies (1:250 rabbit anti-mouse, Cell Signaling Technologies; #9145). Sections were then revealed with 1:400 goat anti-rabbit secondary antibody conjugated to Alexa-568 (Santa Cruz) for 2.5 hours at room temperature. For each group of IF sections, optimal acquisition parameters (photomultiplier gain, intensity offset, and laser excitation intensity) were adjusted at the beginning of each experiment and held constant. For quantification of pSTAT3- and STAT3-immunostained sections, at least five images for each experimental case, were acquired with an Olympus confocal laser-scanning microscope. A 40× objective guaranteeing coverage of the whole extension of each half of the ARC was used. The number of immunoreactive cells was counted manually using ImageJ software (NIH). Cell counts were normalized to the ARC area in each section. The ratio between pSTAT3 and STAT3 was calculated for each animal; the mean of this ratio in H_2_O-CFD, VEH-H_2_O and H_2_O-HFD groups treated with saline was used to calculate percent change in each group, respectively.

### Statistical analysis

GraphPad Prism was used for statistical analysis. Data are expressed as mean ± s.e.m. Comparisons between two groups were made by unpaired 2-tailed Student’s t-test or Mann-Whitney test, as appropriate. Comparisons between three or more groups were made by 1-way ANOVA, 2-way ANOVA or 2-way Repeated Measure (RM) ANOVA followed by Bonferroni *posthoc* or Kruskal-Wallis test followed by Dunns *posthoc* test, as appropriate. A p-value of less than 0.05 was considered to be statistically significant.

## Results

### FLX affects energy balance and leptin sensitivity

**Figure 1:**
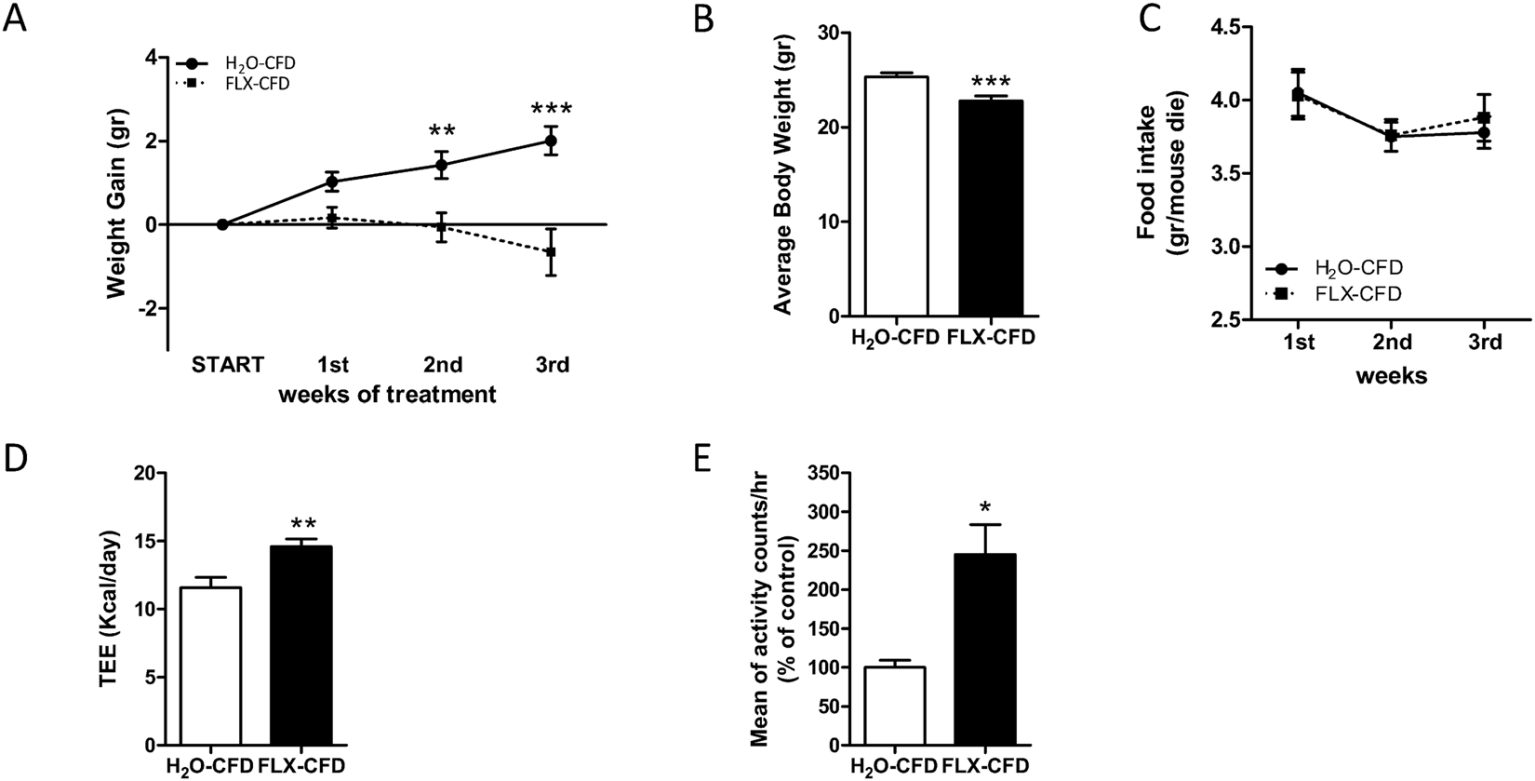
FLX-treated mice show decreased energy balance due to higher energy expenditure. (**A**) Weight gain with respect to start of FLX treatment for H_2_O-CFD and FLX-CFD mice. RM 2-way ANOVA (treatment × time, F(3,117) = 8.54, P < 0.0001; n = 18–23) followed by Bonferroni *posthoc* test, **P < 0.01, ***P < 0.001. **(B)** Body weight at the end of the treatment. Student’s t-test (t = 3.629 df = 45, ***P = 0.0007; n = 21–26). **(C)** Food intake during the 3 weeks of treatment for H_2_O-CFD and FLX-CFD mice (n = 21–26). **(D)** Daily total energy expenditure of H_2_O-CFD and FLX-CFD mice measured by doubly labelled water (DLW) method. Student’s t-test (t = 3.046 df = 20, **P = 0.0064; n = 12–10). **(E)** Infrared locomotor activity monitoring assessed in H_2_O-CFD and FLX-CFD (number of animals = 6; n = 134). Mann Whitney test (*P = 0.0163). Data are presented as mean ± s.e.m.

Adult male mice (p60) were exposed to a chronic treatment with FLX (from now on referred to as FLX-CFD), dissolved in their drinking water. A group of mice drinking normal water served as control (H_2_O-CFD). We first wanted to define how FLX affected the phenotype in terms of energy balance. FLX-CFD gained less weight as compared to H_2_O-CFD (Fig. 1A) and displayed a lower body weight at the end of the treatment (Fig. 1B). We observed a trend towards decrease in epididymal (EPI) and perirenal (PERI) white adipose tissue (WAT) weight in FLX treated mice that may partly account for the lower body weight. Liver, brown adipose tissue (BAT) and subcutaneous (SC) WAT did not weigh significantly different between the 2 groups (Supplementary Figure S1).

Energy balance is the result of energy intake and energy expenditure. Food intake was not significantly different between FLX-CFD and H_2_O-CFD (Fig. 1C). To measure energy expenditure we used the doubly-labeled water (DLW) technique^42–44^, an isotope dilution-based method that can measure free-living energy expenditure. FLX treated animals displayed a significantly higher rCO_2_ and TEE during the 24 hours (Fig. 1D). An important component of energy output is locomotor activity which is higher in FLX-CFD mice as compared to control group (Fig. 1E).

**Figure 2:**
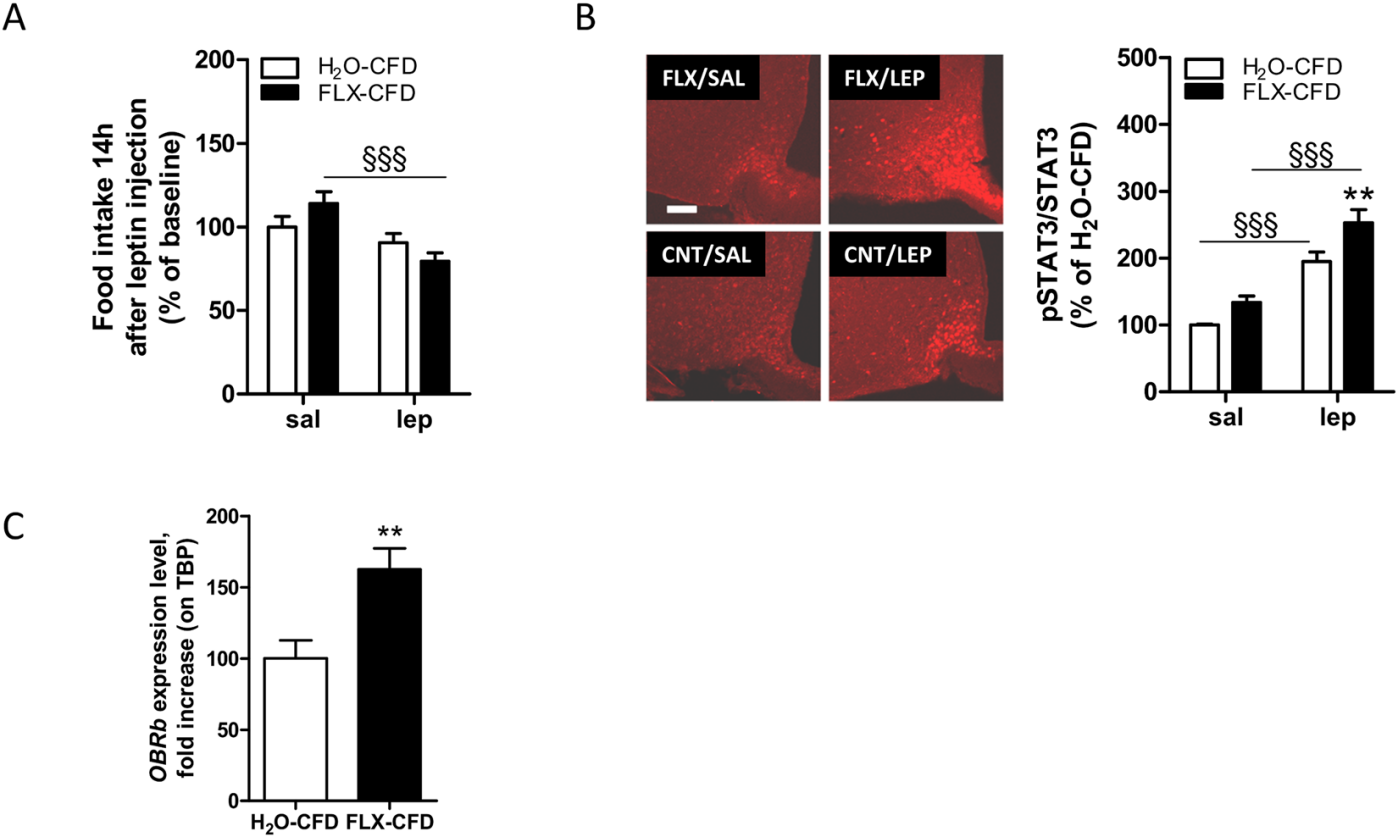
FLX treated mice show enhanced response to acute leptin administration. **(A)** 14-h cumulative food intake in H_2_O-CFD and FLX-CFD mice at baseline and in response to i.p. leptin (3 mg/kg) injection. All data are expressed as % of the mean of the baseline of H_2_O-CFD. RM 2-way ANOVA (treatment x stimulus, F(1,45) = 7.93, P = 0.0072; matching, F(45,45) = 2.77, P = 0.0004; n = 22–25) followed by Bonferroni *posthoc* test, ^§§§^ P< 0.001 *versus* baseline. **(B)** *Left* representative immunofluorescence showing expression of phosphorylated-STAT3 45′ minutes after an injection of saline (sal) or leptin (lep, 3 mg/kg) in the ARC of H_2_O-CFD (CNT) and FLX-CFD (FLX). Scale bar is 100 μm. *Right*, signal for pSTAT3 normalized to that of STAT3 was acquired for H_2_O- and FLX-CFD mice. All data are expressed as % of the mean of the H_2_O-CFD injected with saline. 2-way ANOVA (stimulus effect, F(1,34) = 77.18, P < 0.0001; treatment effect, F(1,34) = 14.06, P = 0.0007; n = 11–8, 10–9) followed by Bonferroni *posthoc* test, **P < 0.01 *versus* H_2_O-CFD, ^§§§^ P< 0.001 *versus* saline. **(C)** *OBRb* mRNA value was determined by quantitative reverse transcription–PCR and standardized to TATA Binding Protein (*TBP*) in the hypothalamus of H_2_O-CFD and FLX-CFD mice. Student’s t-test (t = 3.212 df = 14, **P = 0.0063; n = 8). Data are presented as mean ± s.e.m.

It is well established that leptin positively affects energy expenditure^45^: we then asked whether the leptin system was altered in FLX-CFD mice. Leptin levels were not significantly changed by FLX treatment (2681 ± 584 and 3255 ± 739 pg/ml H_2_O-CFD *vs* FLX-CFD respectively), but when we administered an acute leptin treatment FLX-CFD displayed a more pronounced reduction in food intake compared to controls (Fig. 2A), thus revealing an higher sensitivity to the anorectic effects of the hormone.

Leptin action is mainly achieved through the activation of the JAK2-STAT3 pathway in the ARC: here, the number of pSTAT3-immunoreactive neurons after leptin injection was higher in FLX-CFD mice as compared to H_2_O-CFD (Fig. 2B). In addition, the mRNA abundance for the leptin receptor (*OBRb*) was significantly increased upon FLX treatment (Fig. 2D).

Taken together, these data indicate that FLX treatment inhibits weight gain in adult mice, mainly acting on energy output, and enhances acute hypothalamic leptin sensitivity.

### FLX affects hypothalamic expression of BDNF and modifies WAT expression profile

**Figure 3:**
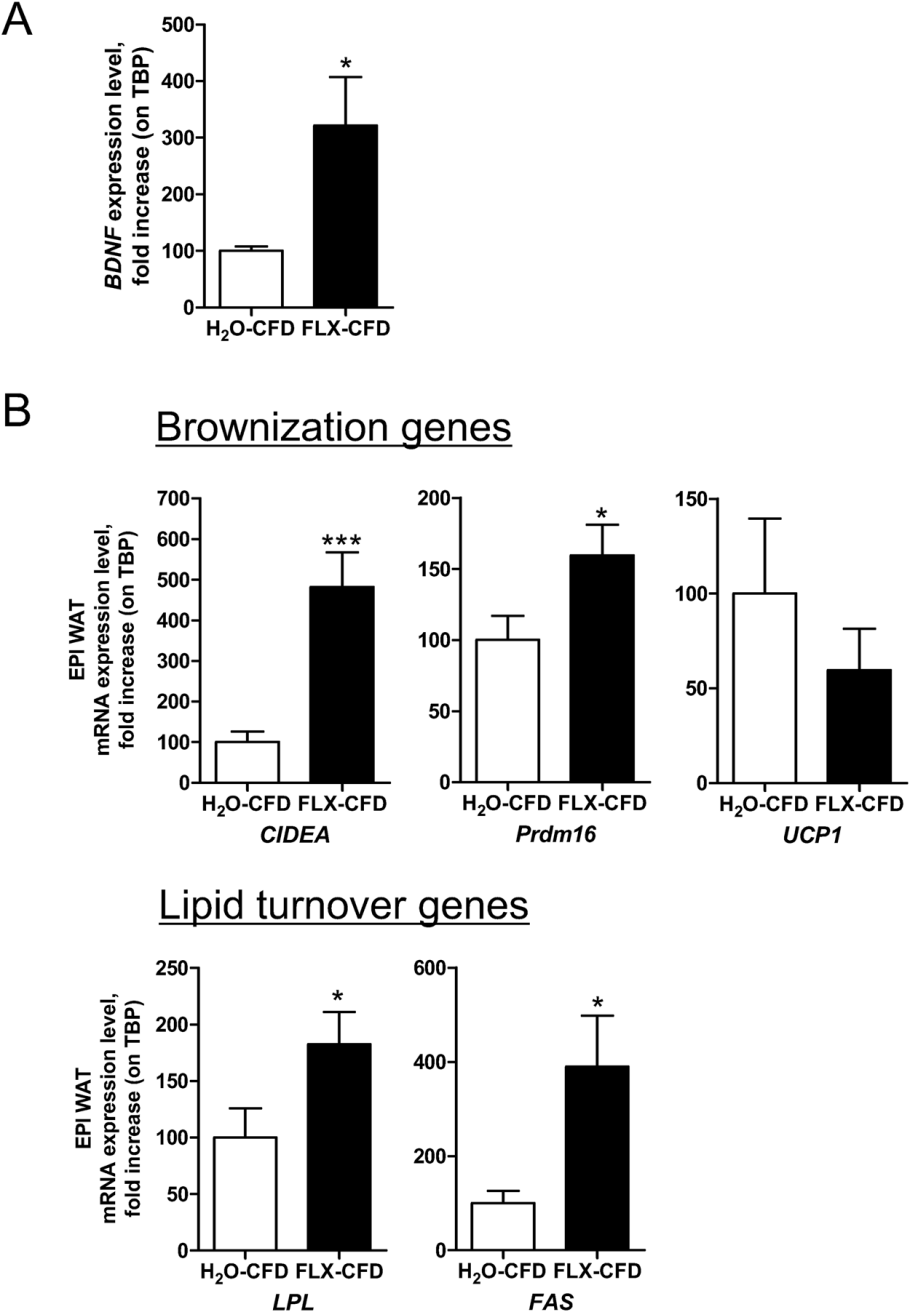
FLX affects hypothalamic expression of BDNF and modifies WAT expression profile. mRNA values were determined by quantitative reverse transcription–PCR and standardized to TATA Binding Protein (*TBP*) in the hypothalamus **(A)** and in the EPI WAT (**B**) of H_2_O-CFD and FLX-CFD. Student’s t-test: (A) *BDNF* (t = 2.577 df = 14, *P = 0.022; n = 8); (B) *CIDEA* (t = 4.099 df = 15, ***P = 0.0009; n = 8–9)*; Prdm16* (t = 2.175 df = 14, *P = 0.047; n = 8); *LPL* (t = 2.151 df = 14, *P = 0.049; n = 8); *FAS* (t = 2.592 df = 14, *P = 0.021; n = 8). Data are presented as mean ± s.e.m.

FLX is known to induce BDNF expression in different brain regions^25,26^: in line with results by Sachs *et al*.^46^, quantitative PCR revealed a significant increase of *BDNF* mRNA abundance in the hypothalamus of FLX-CFD as compared to H_2_O-CFD (Fig. 3A).

Empowerment of BDNF action has been related to alteration of the WAT expression profile^47^ downstream of sympathetic nervous system activation. Indeed, the EPI WAT of FLX-CFD mice exhibited increased expression of Cell Death-Inducing DFFA-Like Effector A (CIDEA) and PR/SET Domain 16 (Prdm16) as compared to controls. CIDEA is highly expressed in murine BAT compared with WAT^48^ and is considered a marker for the emergence of brown-like adipocytes^49^; Prdm16, upstream of the classical BAT marker Uncoupling Protein 1 (UCP1), is necessary for the browning of white fat^50^ and its expression is typical of the so-called beige adipocytes^51^. UCP1 expression is not significantly affected by FLX. We also observed an upregulation of genes involved in lipid turnover including Lipoprotein Lipase (*LPL*) and Fatty Acid Syntase (*FAS*) (Fig. 3B). Some of these changes (*FAS*, P = 0.052 and *CIDEA*) were observed also in the SC WAT of FLX-CFD where an increase of the classical BAT marker *UCP1*, was also found (Supplementary Figure S2).

### FLX in the absence of an intact BDNF pathway

To investigate if there was a relationship between BDNF over expression and the changes described under “FLX affects energy balance and leptin sensitivity“, we treated the knock in Ntrk2^tm1Ddg^/J model, with 1NaPP1, a chemical compound that specifically inhibits TrkB kinase activity in these mutants^33^, and was proved able to cross the blood brain barrier^34^. Untreated animals do not differ from wild type in terms of fertility or viability^33,52^; neither they show difference in physical parameters, food intake or body composition as reported^53^ and directly observed by us (Supplementary Figure S3). Notably, in the absence of TrkB signaling inhibitor, Ntrk2^tm1Ddg^/J retain full BDNF-signaling capabilities^33,54^ and indistinguishable synaptic transmission and plasticity^52^.

2 days before the start of FLX treatment, osmotic minipumps were implanted into the Ntrk2^tm1Ddg^/J model to deliver VEHicle or 1NaPP1; 4 different groups were generated: 1. receiving 1NaPP1 and normal water (1NaPP1-H_2_O); 2. receiving 1NaPP1 and FLX (1NaPP1-FLX); 3. receiving vehicle and normal water (VEH-H_2_O) and, finally, 4. receiving vehicle and FLX (VEH-FLX).

#### Energy balance

**Figure 4:**
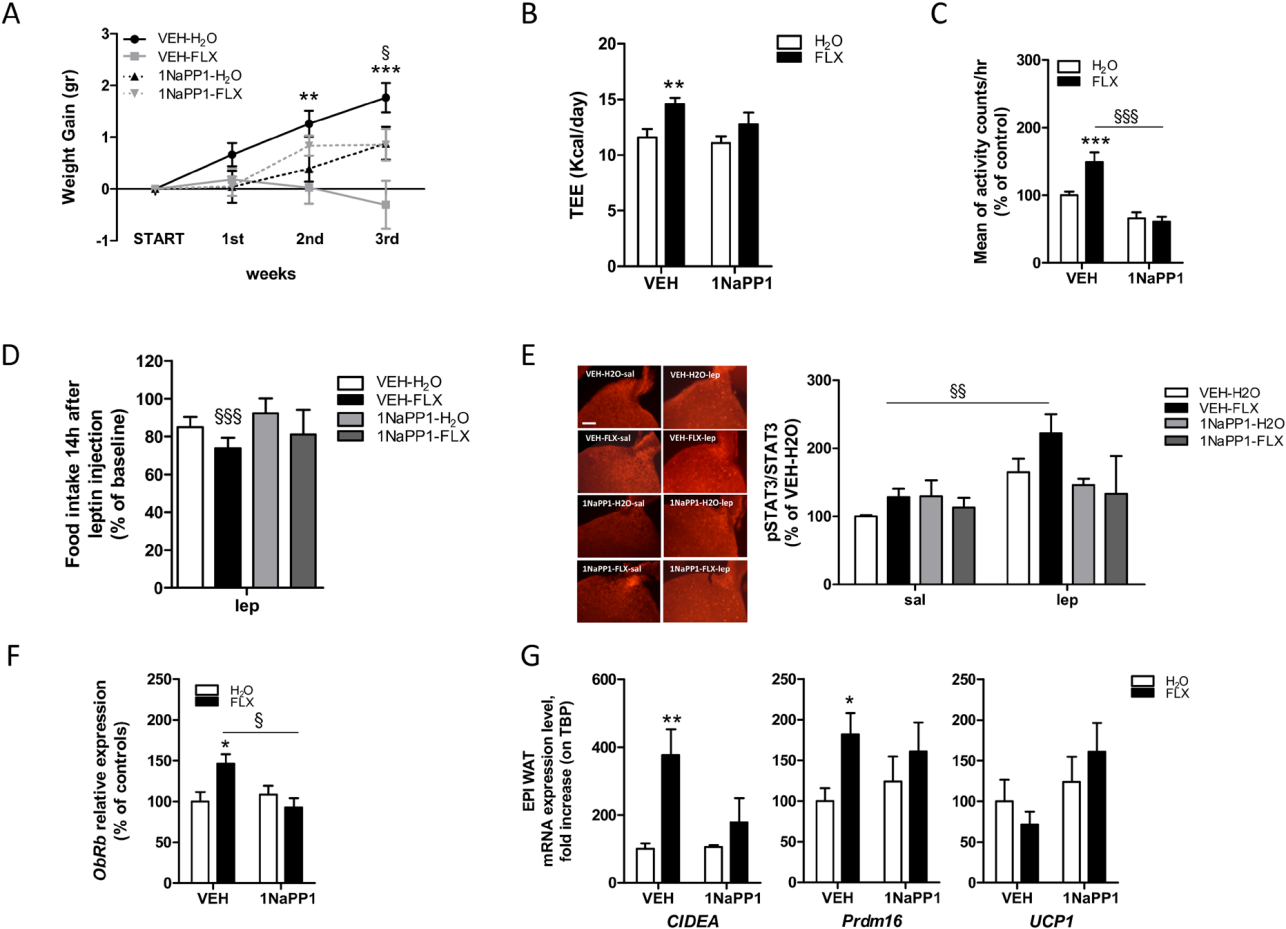
FLX effects in the absence of an intact BDNF pathway. (**A**) Weight gain with respect to start of FLX treatment for VEH-H_2_O (drinking water and with vehicle minipumps, n = 16), VEH-FLX (drinking fluoxetine, and with vehicle minipumps, n = 30), 1NaPP1-H_2_O (drinking water and with 1NaPP1 minipumps, n = 11) and 1NaPP1-FLX (drinking fluoxetine and with 1NaPP1 minipumps, n = 10). RM 2-way ANOVA (treatment × time, F(9,216) = 3.75, P = 0.0002) followed by Bonferroni *posthoc* test, **P < 0.01, *** P < 0.001 VEH-FLX *versus* VEH-H_2_O; ^§^P < 0.05 VEH-FLX *versus* 1NaPP1-FLX. **(B)** Daily total energy expenditure of the indicated groups measured by doubly labelled water method. 2-way ANOVA (treatment, F(1,27) = 7.37, P = 0.011; n = 12–10, 5–4) followed by Bonferroni *posthoc* test, **P < 0.01 *versus* H_2_O. **(C)** Infrared locomotor activity monitoring assessed in the indicated groups as average activity counts per hour during the day (number of animals = 9; n = 192). 2-way ANOVA (treatment × compound, F(1,1054) = 6.06, P = 0.014) followed by Bonferroni *posthoc* test, ***P < 0.001 *versus* H_2_O, ^§§§^P < 0.001 *versus* VEH. **(D)** Percentage of food intake variation from baseline 14-h after i.p. leptin (3 mg/kg) injection in VEH-H_2_O (n = 20), VEH-FLX (n = 22), 1NaPP1-H_2_O (n = 5) and 1NaPP1-FLX (n = 7) mice. RM 2-way ANOVA (stimulus effect, F(1,50) = 14.47, P = 0.0004; matching, F(50,50) = 2.67, P = 0.0004) followed by Bonferroni *posthoc* test, ^§§§^P < 0.001 *versus* baseline. **(E)** *Left*, representative immunofluorescence showing expression of pSTAT3 45’ after an injection of saline (sal) or leptin (lep, 3 mg/kg) in the ARC of VEH-H_2_O (n = 12), VEH-FLX (n = 12), 1NaPP1-H_2_O (n = 3) and 1NaPP1-FLX (n = 3) mice. Scale bar is 100 μm. *Right*, signal for pSTAT3 normalized to that of STAT3 was acquired for the indicated groups. All data are expressed as % of the mean of the VEH-H_2_O mice injected with saline. 2-way ANOVA (stimulus effect, F(1,48) = 6.55, P = 0.014) followed by Bonferroni *posthoc* test, ^§§^P < 0.01 *versus* saline. **(F)** *OBRb* mRNA value was determined by quantitative reverse transcription–PCR and standardized to TATA Binding Protein (*TBP*) in the hypothalamus of the indicated groups. 2-way ANOVA (treatment × compound, F(1,29) = 4.39, P = 0.045; n = 12–12, 5–4) followed by Bonferroni *posthoc* test, *P < 0.05 *versus* H_2_O, ^§^P < 0.05 *versus* VEH. **(G)** mRNA values were determined by quantitative reverse transcription–PCR and standardized to TATA Binding Protein (*TBP*) in the EPI WAT of VEH-H_2_O,VEH-FLX, 1NaPP1-H_2_O and 1NaPP1-FLX mice. 2-way ANOVA: *CIDEA* (treatment effect, F(1,28) = 4.49, P = 0.043; n = 10–14, 5–4); *Prdm16* (treatment effect F(1,20) = 4.82, P = 0.04; n = 7–8, 5–4); *UCP1* (ns; n = 12–12, 5–4) followed by Bonferroni *posthoc* test, *P < 0.05, **P < 0.01 *versus* H_2_O. Data are presented as mean ± s.e.m.

The negative effect of FLX on weight gain was attenuated in mice with a blunted BDNF function (1NaPP1-FLX, Fig. 4A). The 4 groups showed similar food intake (not shown). We then asked whether 1NaPP1 administration reverses the positive effect of FLX on energy output observed in wild type mice. This is indeed the case, as established by DLW method which revealed that TEE of 1NaPP1-FLX mice does not significantly differ from controls (Fig. 4B). Finally, the increase in locomotor activity that we observe in VEH-FLX disappears in 1NaPP1-FLX mice (Fig. 4C).

#### Leptin sensitivity

Upon 1NaPP1 administration, FLX loses its capacity to amplify the response to leptin as represented in Fig. 4D, where 1NaPP1-FLX mice fail to show the leptin-dependent drop in food intake observed in VEH-FLX mice. In line with these data, STAT3 activation (pSTAT3/STAT3) in 1NaPP1-FLX injected with leptin does not differ from leptin injected VEH-H_2_O. On the other hand, leptin-injected VEH-FLX show a significant increase in pSTAT3 as compared to leptin stimulated VEH-H_2_O (Fig. 4E). Similarly, the expression of *OBRb* in the hypothalamus returns to the level of controls (VEH-H_2_O) in 1NaPP1-FLX mice (Fig. 4F).

#### WAT gene expression

Interestingly, when BDNF action is blunted by 1NaPP1 the changes in WAT gene expression induced by FLX in wild type mice are attenuated; indeed *CIDEA* and *Prdm16*, upregulated in the EPI WAT of VEH-FLX, are not different from VEH-H_2_O in 1NaPP1-FLX mice (Fig. 4G). A similar trend can be observed for *UCP1* and *CIDEA* in the SC WAT (Supplementary Figure S4).

In aggregate, these data indicate that FLX effects on energy balance and leptin sensitivity are at least partly mediated by an intact BDNF pathway.

### Fluoxetine and high fat diet

The results described above elucidated important mechanisms underlying the control of energy balance by FLX; we next wanted to investigate the effect of the drug during weight gain due to excessive energy intake.

To this end, mice already fed with high fat diet (HFD) for 6 weeks were divided into 2 groups with similar body weight and weight gain since the start of HFD: 1. H_2_O-HFD, kept on HFD for a total of 9 weeks which served as a control group; 2. FLX-HFD, which received FLX treatment from week 7 through week 9, since HFD start.

#### Energy balance

**Figure 5:**
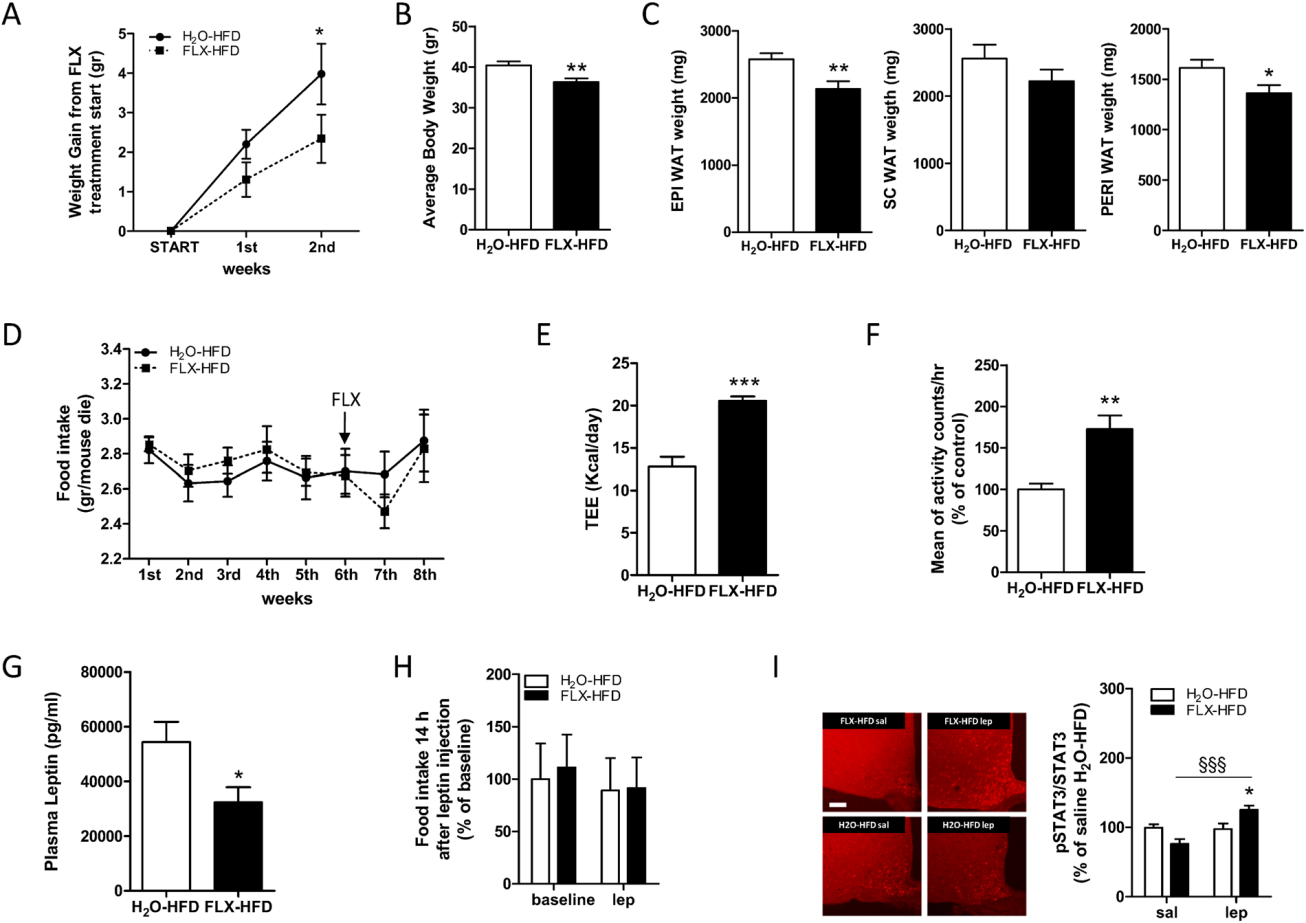
FLX acts on energy balance and leptin sensitivity in animals exposed to high fat diet. (**A**) Weight gain with respect to start of FLX treatment for H_2_O-HFD and FLX-HFD mice. RM 2-way ANOVA (time effect, F(2,54) = 30.18, P < 0.0001; matching, F(27,54) = 1.85, P = 0.027; n = 24–27) followed by Bonferroni *posthoc* test, *P < 0.05. **(B)** Body weight at the end of the treatment in H_2_O-HFD and FLX-HFD mice. Student’s t-test (t = 3.02 df = 34, **P = 0.0048; n = 18). **(C)** Weight of white fat depots (EPI, SC and PERI) in H_2_O-HFD and FLX-HFD mice. Student’s t-test (EPI WAT t = 2.932 df = 31, **P = 0.0063; PERI WAT t = 2.169 df = 31, *P = 0.038; n = 15–18). **(D)** Food intake in H_2_O-HFD and FLX-HFD; the arrow indicates the start of FLX treatment and each point represents a daily average per week (n = 24–27). **(E)** Daily total energy expenditure of H_2_O-HFD and FLX-HFD mice measured by doubly labelled water method. Student’s t-test (t = 5.885 df = 17, ***P < 0.0001; n = 10–9). **(F)** Infrared locomotor activity monitoring assessed in H_2_O-HFD and FLX-HFD mice (number of animals = 6; n = 100). Mann Whitney test (**P = 0.0073). **(G)** Plasma leptin in H_2_O-HFD and FLX-HFD mice. Student’s t-test (t = 2.428 df = 15, *P = 0.028; n = 8–9). **(H)** 14 hours cumulative food intake in H_2_O-HFD and FLX-HFD mice at baseline and in response to i.p. leptin (3 mg/kg) injection (n = 11–7). **(I)** *Left*, representative immunofluorescence showing expression of pSTAT3 45’ after an injection of saline (sal) or leptin (lep, 3 mg/kg) in the ARC of H_2_O-HFD and FLX-HFD. Scale bar is 100 μm. *Right*, signal for pSTAT3 normalized to that of STAT3 was acquired in H_2_O-HFD and FLX-HFD mice. All data are expressed as % of the mean of the H_2_O-HFD injected with saline. 2-way ANOVA (treatment × stimulus, F(1,12) = 14.78, P = 0.0023; n = 3) followed by Bonferroni *posthoc* test, *P < 0.05 *versus* H_2_O, ^§§§^P < 0.001 *versus* saline. Data are presented as mean ± s.e.m.

As shown in Fig. 5A FLX-HFD gained significantly less weight as compared to controls and displayed lower body weight at the end of the treatment (Fig. 5B); fat depots (Fig. 5C) were also smaller in FLX-HFD as compared to H_2_O-HFD. No difference was observed in the weight of liver or BAT (data not shown). FLX treatment did not significantly alter food intake (Fig. 5D), but energy expenditure was greatly increased by FLX in HFD mice (Fig. 5E). Locomotor activity was induced by FLX in HFD animals (Fig. 5F).

#### Leptin sensitivity

In spite of the similar food intake found in the 2 groups, leptin was significantly lower in FLX-HFD (Fig. 5G) as compared to H_2_O-HFD, indicating that the response to endogenous hormone is higher. When recombinant leptin was administered acutely, nor H_2_O-HFD, neither FLX-HFD significantly diminished their food intake (Fig. 5H). An interesting finding, however, emerged when we evaluated the extent of STAT3 phosphorylation in the ARC of FLX-HFD and H_2_O-HFD mice in response to leptin injection. The latter did not show any significant increase in the number of pSTAT3-immunoreactive cells in comparison to H_2_O-HFD mice injected with saline (Fig. 5I), thus confirming the expected pattern of cerebral leptin resistance. Conversely, leptin-injected FLX-HFD displayed a significant activation of the STAT3 pathway. No difference was found in the hypothalamic expression of *OBRb*, whereas a trend towards higher *BDNF* expression, albeit not significant, was found in FLX-HFD versus H_2_O-HFD (Supplementary Figure S5).

Altogether these findings indicate that upon HFD, FLX is able to reduce weight gain acting on energy expenditure and to attenuate the effect of obesity on blunting leptin signaling.

## Discussion

During the 90 s’ many clinical trials employed FLX to induce weight loss with initially successful outcomes. However, these improvements were in most cases lost after the first months of treatment. In the long run patients tended in fact to regain weight while continuing FLX therapy^32,55^. This has dampened the enthusiasm for the use of this drug for obesity therapy, even if in obese patients with a depression spectrum FLX is still being used. These studies were never rethought in the light of the seminal findings of the past 20 years on energy homeostasis and the underlying intense crosstalk between periphery and central nervous system. By filling this gap we herein found new ground to explain past clinical observations as well as introducing the concept that FLX is able to impinge on crucial mechanisms for the homeostatic control of energy.

Chronic FLX to male mice herein resulted in diminished weight gain, increased acute central response to leptin and higher hypothalamic BDNF expression. The observed reduction in the weight gained over FLX treatment was observed both in lean and obese FLX treated animals and can be partly explained by the increased total energy expenditure. This may take place through two different mechanisms: 1. a direct action, suggested by its capacity to induce the hypothalamic adrenal pituitary axis^56^, a process associated with a higher mobilization of energy stores^57,58^ this being consistent with FLX positive effect on resting energy expenditure in obese women^59^ and 2. through the enhanced sensitivity to leptin, long known to promote energy expenditure. In fact, in the leptin deficient hypoactive *ob/ob* model leptin administration normalizes locomotor as well as thermogenic activity^60^. Importantly, STAT3 mediates such leptin action as gain or loss of function of this pathway result in increased or decreased activity respectively^61,62^. The effects of leptin on locomotor cage activity occurs since the beginning of a leptin treatment and within the physiological range of concentrations, thus well before any observed effect on weight loss^63,64^. In the lean FLX-treated mouse we found increased sensitivity to the acute anorectic and signaling effects of the hormone, which may be enough to promote increased energy expenditure, without reaching the threshold to affect the leptin axis that regulates feeding behavior: our data, in line with others’^65^, indicate in fact that FLX does not significantly change food intake in mice under normal diet regimen. Interestingly, in the FLX HFD mice no anorectic response is observed upon acute leptin stimulus, but STAT3 activation is preserved and correlates with higher energy expenditure. Saturation of STAT3 signaling, totally in place in our untreated HFD mice, is one of the reasons accounted for to explain leptin resistance in obesity^66^ and FLX seems to at least partly counteract this phenomenon. Data on the obese model holds interesting translational implications, as in this case, increased sensitivity to an acute injection is also accompanied by a chronic impact on the endogenous leptin system: in fact food intake was similar to untreated HFD, but corresponded to lower levels of the hormone. We previously reported increased chronic sensitivity to the hormone in the environmentally enriched lean mouse^15^, a condition that is not reproduced in the FLX treated lean mouse, but that seems unmasked by the metabolic dynamics associated to HFD.

Recent work conducted on rats indicates that FLX is able to enhance a specific component of energy expenditure, *i.e*. the thermogenic capacity of WAT, by increasing mitochondrial function^67^. Indeed, in the present study FLX-CFD mice showed some alterations of their WAT expression profile with an enhanced expression of genes typically modulated in the trans-differentiation between white and brown adipocytes (brite cells)^68^: under FLX treatment *CIDEA* and *Prdm16* are in fact upregulated in the EPI WAT, while SC WAT shows a significant upregulation of *CIDEA* and *UCP1*. Prdm16 is an early determinant of brownization, while UCP1 is considered a marker of terminal differentiation^68^. This being considered, SC WAT under FLX seems more advanced in the trans-differentiation process as compared to EPI WAT. Recent findings show a higher number of cells able to become beige in SC as compared to EPI WAT, and this may partly account for the difference herein observed^69,70^.

It is important to emphasize that brownization oriented changes in WAT gene expression herein found correlate with an induction of hypothalamic BDNF expression, and artificially hindering of this neurotrophin function abolishes response to FLX treatment. These data are in line with the elegant work conducted by Cao^47^ in the environmentally enriched mouse model, for which he postulates and demonstrates a connection between induction of hypothalamic BDNF expression, activation of the sympathetic nervous system and changes in WAT expression profile towards brownization.

This work can be summarized in 3 main concepts: the first is that leptin sensitivity can be modulated by this antidepressant in adulthood, even upon obesity. Second, BDNF plays an important role in mediating FLX action on energy homeostasis. BDNF positive action on energy output has recently emerged: increased energy expenditure is in fact reported for mice undergoing local hypothalamic BDNF infusion^71,72^ and mice carrying deletion in hypothalamic TrkB in the hypothalamus show diminished locomotor activity and resting energy expenditure^73^. Our FLX model resulted in an upregulation of BDNF, that correlates with increased energy expenditure, an effect greatly attenuated when the BDNF receptor is not functional. We discussed above the possibility that an increased sensitivity to leptin is implied in the increased energy output. This leads us directly to the third concept/implication disclosed by the present work, *i.e*. the relationship between FLX, hypothalamic leptin sensitivity and an intact BDNF pathway. Our data indicate the presence of an important crosstalk between mechanisms driving leptin sensitivity in the ARC and BDNF function, this being in line with the observation that hypothalamic specific deletion of TrkB results in significant loss of leptin sensitivity^73^. Further investigations will be necessary to establish whether this takes place through a direct molecular interference or downstream of a remodeling action of BDNF on synaptic plasticity. Indeed, BDNF induction has been implied as a key step of antidepressants therapeutical action, for its capacity to enhance synaptic plasticity, thus providing a substrate for behavioral changes^74^. FLX makes no exception in this regard, and upon BDNF haploinsufficiency its action on behavioural model of anxiety and depression is lost^75,76^.

In addition, findings of the present study provide ground for speculation when we try to reconcile them with the transient effect of FLX as an obesity therapy: changes in gene expression and activation herein described likely persist throughout the treatment and possibly beyond, but become insufficient for a phenotypic outcome during the treatment of obese humans because thresholds for effective stimulation might increase. These changes could in any case constitute a sort of FLX epigenetic imprint, as reported for other brain regions and functions^3,77^ able to confer a different susceptibility to metabolic or pharmacological challenges.

It’s worth mentioning that the present study employs male mice only, whereas in the human population FLX is used as a therapy for major depression in both genders and more widely in women that show a higher prevalence for this disorder^78^. This obviously poses some limitations when concluding on the present findings and the clinical use/outcome of FLX.

In conclusion, we substantiated the long known and debated impact of the SSRI FLX on energy balance with two novel underlying mechanisms, namely its capacity to induce hypothalamic expression of BDNF and to enhance central leptin sensitivity. Importantly, the two mechanisms are interrelated and the component that results predominantly affected is energy output.

Further, we demonstrated that it is possible to modulate the threshold for leptin sensitivity in adult mice by a pharmacological approach based on an “enviromimetic” molecule.

## Electronic supplementary material

Supplementary Material
