# Supplementary material for: The antidepressant fluoxetine acts on energy balance and leptin sensitivity via BDNF
Source: Sci Rep. 2018 Jan 29;8:1781. doi: 10.1038/s41598-018-19886-x (PMC5789051; doi:10.1038/s41598-018-19886-x)
Supplement: Supplementary file 1 — Supplementary Material [file 41598_2018_19886_MOESM1_ESM.doc]

**The antidepressant fluoxetine acts on energy balance and leptin sensitivity via BDNF.**

Gaia Scabia^1,2^, Ilaria Barone^2,3^, Marco Mainardi^5*^, Giovanni Ceccarini^1^, Manuela Scali^5^, Emma Buzzigoli^4^, Alessia Dattilo^1^, Paolo Vitti^1^, Amalia Gastaldelli^4^, Ferruccio Santini^1^, Tommaso Pizzorusso^5,6^, Lamberto Maffei^5^, Margherita Maffei ^1,4^

1. Obesity Center at the Endocrinology Unit, Department of Clinical and Experimental Medicine, Via Paradisa 2, 56126 Pisa, Italy

2. Dulbecco Telethon Institute

3. Laboratory of Neurobiology, Scuola Normale Superiore, Piazza dei Cavalieri 7, 56100 Pisa, Italy

4. Institute of Clinical Physiology, Italian National Research Council, Via Moruzzi 1, 56124 Pisa, Italy

5. Institute of Neuroscience, Italian National Research Council, Via Moruzzi, 56124 Pisa, Italy

6. Dipartimento NEUROFARBA, Università di Firenze, via di San Savi, 26, 50235 Firenze, Italy

* Current address: Università Cattolica, Lgo Francesco Vito, 1, 00168 Roma, Italy

**Correspondence:** [**m.maffei@ifc.cnr.it**](mailto:m.maffei@ifc.cnr.it)

**Key words:** leptin sensitivity, obesity, brain derived neurotrophic factor, hypothalamic arcuate, energy expenditure.

**Running title:** Fluoxetine and mechanisms of energy homeostasis**.**

**Supplementary Information (SI)**


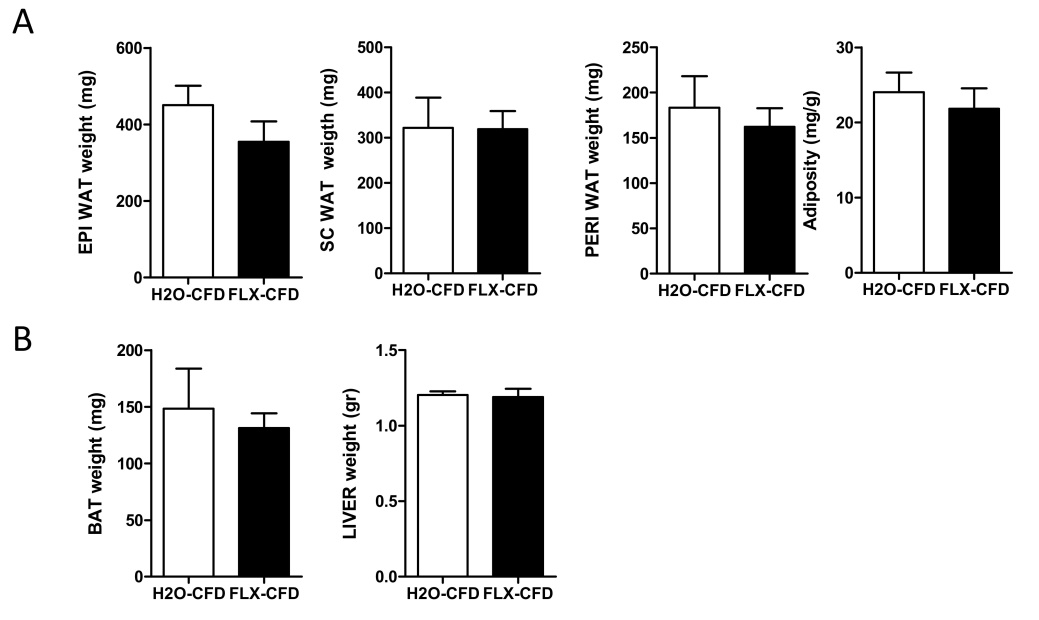


**Supplementary Figure S1. (A)** Weight of white fat depots (EPI, SC and PERI WAT) and adiposity (sum of weight of white fat depots/body weight) of H_2_O-CFD and FLX-CFD mice (n=6-9). **(B)** Bat and liver weight of H_2_O-CFD and FLX-CFD mice (n=6-9). Data are presented as mean ± s.e.m.

Brownization genes

Lipid turnover genes

**Supplementary Figure S2. Analysis of subcutaneous WAT gene expression in adult mice.** mRNA values were determined by quantitative reverse transcription–PCR and standardized to TATA Binding Protein (*TBP*) in the SC WAT of H_2_O-CFD and FLX-CFD mice. Student’s t-test: *CIDEA* (t=2.278 df=16, *P=0.037; n=8-10); *Prdm16* (ns; n=9); *UCP1* (t=2.449 df=15, *P=0.027; n=8-9); *LPL* (ns; n=9-10); *FAS* (ns; n=9). Data are presented as mean ± s.e.m.

**Supplementary Figure S3. Physical parameters, body weight and food Intake in CNT (WT) and Ntrk2^tm1Ddg^/J.** Animals were sex-age matched and food intake was monitored for 3 weeks (n=6-8). Data are expressed as mean ± s.e.m.

**Supplementary Figure S4. Analysis of subcutaneous WAT gene expression for UCP1 and CIDEA in the absence of an intact BDNF pathway.** mRNA values were determined by quantitative reverse transcription–PCR and standardized to TATA Binding Protein (TBP) in the SC WAT of VEH-H_2_O (n=13), VEH-FLX (n=13), 1NaPP1-H_2_O (n=5) and 1NaPP1-FLX (n=4) mice. Data are presented as mean ± s.e.m.


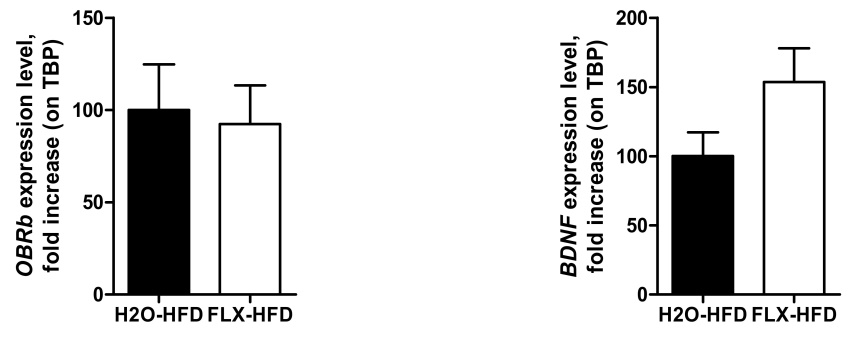


**Supplementary Figure S5. Analysis of hypothalamic gene expression in HFD mice.** mRNA values were determined by quantitative reverse transcription–PCR and standardized to TATA Binding Protein (*TBP*) in the hypothalamus of H_2_O-HFD and FLX-HFD (n=16). Data are presented as mean ± s.e.m.
